# Supplementary material for: Assessing a Video-Based Intervention to Promote Parent Communication Strategies with a Deaf Infant: A Feasibility and Acceptability Study
Source: J Clin Med. 2022 Sep 7;11(18):5272. doi: 10.3390/jcm11185272 (PMC9505164; doi:10.3390/jcm11185272)
Supplement: Supplementary file 1 [file jcm-11-05272-s001.zip › Supplementary Materials S1_Study1VideoScript.pdf]

Supplementary Materials S1: Study 1 Video Script

Welcome to ‘Communicating with Your Baby’, a video about ways to communicate with your baby when having fun in your everyday routine.

Even before they produce their first words, babies are learning how to communicate with other people. All children, hearing and deaf, learn in different ways. For deaf children, some parts of learning to communicate can be difficult. This video is about the things that we can do to make it easier. It is all about ways to communicate with your baby and ways to encourage their communication. The main ideas about communicating with babies are the same for both deaf and hearing babies, but for a baby with hearing loss, we need to make some adaptations. If some of these adaptations are new to you, and you would like to continue using them, soon they will feel like a natural part of your everyday communication.

Around their 1st birthday is an exciting time, as babies’ communication skills really improve as they start to want to share what they are interested in with the people around them. A great way to encourage your baby’s developing communication skills is by being in joint attention with your baby. This is when an adult and baby are sharing attention together when focusing on the same object or event.

For example, this mother and baby are looking at a picture book together. Though they have the book in front of them, they may not be paying attention to the same thing. The baby may be looking at the picture of a cow whilst the mother is reading about the horse. So, how do you know you are attending to the same thing? If your baby were to look at the cow, look at you, point to the cow then look back to check you were also looking at the cow, then you are both attending to the cow and more importantly, you both know that the other is attending to the same object. Now you are in joint attention.

Being in joint attention is a great way for babies to learn language and to further develop their communication skills and social skills. Through joint attention, babies can learn about communication.

The tips in this video are things that you can try to make it easier to be in joint attention with your baby. These tips apply no matter what language you have chosen to use with your child.

With the support of your local practitioners, every parent will come to a decision about which language they prefer to use. Some choose speech and natural gestures that we use in everyday communication, some choose to sign, and some choose to use a combination of both. This video is designed for all parents no matter which language you have chosen to use.

The rest of this video will present tips in four steps:

1. Getting down to eye level
2. Watching what your baby is focussing on
3. Attracting your baby's attention
4. Responding to your baby's attempts to communicate with you

These tips are to help make it easier to share attention with your baby when focusing on the same object or event – they will make it easier to be in joint attention.

1. **<u>Getting down to eye level</u>**

Eye contact is an important part of communication with all children, and for a deaf child this is even more so as they need to be able to look at you easily whilst you play together. Getting down to your child's eye level is also great for when you use language. At this level, it will be easier for your child to see your lip patterns and signs, and it will be easier for your child to hear you if they are using hearing technologies, such as cochlear implants or hearing aids. It will also be easier for your baby to see your facial expressions and natural gestures. So the first tip is:

Get down to their eye level

In this photo, you can see that the little boy can see his mum's face and body easily. The idea is to get in just the right position so that your baby can see your face and hands and can easily switch between looking at what they are playing with and looking at you. Depending on your baby's hearing status and if they are using hearing technologies, they will also find it easier to hear you. As well as being in your baby's line of vision, if you can, try to make sure there is light on your face to help your baby see you clearly. Being where your baby can see you easily will make it easier for you and your baby to achieve and maintain joint attention, and have fun together.

The key thing to remember is the importance of visual cues and making eye contact. Your child will know that you are sharing interest in something with them by being able to see

your face. Depending on your baby's hearing status and if they are using hearing technologies, they will also be likely to hear you. Also, to understand what you are saying and/or signing your child needs to look at you.

2. **<u>Watching what your baby is focussing on</u>**

As well as getting down to your child's eye level, it can be a good idea to take a moment to sit back and watch whether something has caught your baby's attention, try to follow their lead. Communication can be more rewarding for babies if it is about what they are already interested in. We can usually tell that a baby is interested in something if....

…They are looking at it
…They are playing with it, touching it, or moving it around
…Sometimes, they may point at an object, or try to show or give it to you

The second tip is very simple:

Take the time to notice what your baby is focusing on

Once you have noticed what your baby is focusing on, you will be able to join in, perhaps by looking at the same thing with them, joining their play, and talking to them about what has already got their attention – this is joint attention.

In this picture, you can see the little boy is playing with a star shaped toy. This is a great opportunity for his mum to start to talk about what he is focussing on, and can also join in his play by helping him find where the star shape fits into his toy.

If your baby tries to tell you something about what they are interested in by making speech-like sounds, pointing, or other gestures, you can respond to their attempts using warm facial expressions and by talking or signing about their interest. Also, your baby may need a little more time to process what you are saying and/or signing, so take your time and use lots of repetition.

For your baby to know you are engaged with them and sharing attention with them, they will need to be able to see your face. When you respond to your baby's interest or attempts to

communicate and your baby doesn't notice that you want to tell them about something, there are a few things you can do to get their attention.

3. **Getting your child's attention**

When getting your child's attention, be where your baby can see you clearly. If your baby is using hearing technologies, you will have been given guidance to get your baby's attention using sound first, for example, by calling their name. This guidance will have been given to develop the use of their hearing technologies. When calling their name, be near your baby and clear when you speak so that your baby can see your face and mouth clearly, and can also process sound depending on their hearing status and whether or not they are using any hearing technologies. If this doesn't work, or if your child does not wear hearing technologies, you can use other methods to get your child's attention. These are…

…Moving into your baby's line of vision
…Waving in your baby's line of vision
…You can also use touch. Deaf people use touch to get attention much more than hearing people. It can be a great way of letting your child know you want to talk to them.

Tip 3 is: Get your baby's attention. Call their name near and clear. If your child does not use any hearing technologies and if sound often does not work, then try touch, waving, or moving into their visual space.

In this video, you will see a mum using touch to get her little boy's attention…
[Video Clip 1]

To get attention with touch, you can tap your baby on the shoulder, the arm, and even the leg when they are sitting. Try not to tap your baby if you are behind them where they can't see you. This could startle your baby and will make it harder for them to respond by looking at you.

To get your baby's attention by waving, try to wave your hand close to their face and in their field of vision. This is another really good visual cue to let your baby know you want to talk or sign to them. Deaf people do this a lot too.

Get down to your baby's eye level when using these attention getting methods so it's not a strain for them to look to you. When your child looks to you in response, show that you are pleased, to encourage them to do it again. When your baby is looking at you, try not to get distracted so you can maintain eye contact.

It is worth being persistent when getting your baby's attention.
[Video Clip 2]

In this clip, the mum taps her son Jimmy, and also says his name until he looks to her. When she has his attention, she is then animated in her response to him and points to a picture of a bear saying and signing, 'bear'.

If you are using touch, sometimes you may need to tap firmly. You can also alternate between sound, touch, and waving in your baby's field of vision until they look at you. How you use these attention getters will depend on the hearing status of your baby, and whether or not they are using hearing technologies. If you have been advised to use sound first, try using sound to get your baby's attention by being **near** and **clear**. If this doesn't work, you can try touch, waving, or alternating between all three.

Sometimes these attention getters won't always work. Even when you persistently try to get your baby's attention they may not look at you. This is OK too as with all babies, sometimes it is difficult to get their attention, especially when they are looking at something else, as can be seen in the next example:
[Video Clip 3]

Here Jimmy's mum tried to get his attention but he was looking at something else and moving away from his mum towards it. As he became interested in something else, this is a really great opportunity to watch what he is focusing on and share attention in what he is interested in.

The main idea is to give these attention getters a try whenever you would like to tell your baby something about what they are interested in. Your baby may not look at you the first 1 or 2 times but if you persist, they will be more likely to look at you. Obviously, this won't

always work and that’s fine. When being persistent at getting your child’s attention, alternate sound, tapping, waving, and even bringing your face into your baby’s field of vision.

If you are signing, you can even put the sign in front of your baby so they will see it and will be more likely to look to you. When they do look to you, you can repeat the sign a few times.

No matter which method you use to get your child’s attention, if they respond by looking at you, try to be animated with excited facial expressions. The idea here is that babies will often need a visual cue that shows you enjoy sharing attention with them. This can be very rewarding for your child. The main aim is for them to learn to look to you more often, even when you are not trying to get their attention. Our next section is all about tips for responding to your baby.

4. **Responding to your baby**

As well as getting your baby’s attention, it is important to be on the lookout for when they are trying to get yours, and to respond quickly. They might spontaneously look at you and make eye contact, or use gestures like pointing to direct your attention to the object or event that they are interested in. This is a great opportunity to join in and share in your baby’s interest. You can show your engagement by looking back and forth between your baby and the object they are playing with, and by having animated facial expressions to show your interest and excitement visually. So the fourth tip is:

Respond to your baby’s attempts to communicate with you

Research suggests that all children find it very rewarding when parents respond to their attempts to communicate. You can also talk or sign to them about what they are focussing on.

In this next clip, the little boy sees a picture of a rhinoceros on his book. He then points to his nose and looks up to his mum to make eye contact. He then pulls at the tab on the book with the picture of the rhino and points to his nose again. His mum notices and in an animated way she says and signs, ‘rhino!’, ‘rhino!’. Here, Jimmy and his mum are sharing attention together as they focus on the picture of a rhinoceros; they are in joint attention and having fun together.

[Video Clip 4]

Talking or signing about what your child is interested in is a great way for your child to learn new words. For example, this little boy is looking at the washing machine. If his parent says or signs, ‘washing machine’ while he is interested in it, there is a good chance he would start to learn this word. But, if, while he is looking at the washing machine, his parent is preparing lunch and getting his attention to talk or sign about lunch, there is less chance he would learn the words for lunch foods, since he is not showing an interest in them at that time.

When you are talking or signing about an object or event, use facial expressions to support what you are saying or signing. So if you are saying or signing, “That’s right!” nod your head when you are doing this with lots of smiling.
[Video Clip 5]

When we talk to babies, we naturally speak in a different way. We make changes to the way we talk to them by speaking slower, in a higher pitch, emphasising words and phrases, and speak in a more rhythmic way. We use a more lively and child friendly voice.
[Video Clip 6]

Babies prefer this way of speaking as they find it more interesting. It catches their attention and helps to develop their communication skills. So when you speak to your baby, try using a more child friendly voice.

If you are signing with your baby, you can make similar changes by signing more slowly, making your signs bigger, and placing the signs near the object you are signing about. You can even guide your baby to make the sign. In the example where Jimmy’s mum says and signs ‘bear’, she also guides Jimmy to make the sign, ‘bear’. If you often sign and talk at the same time like Jimmy’s mum, you can bring these tips together.

Putting it all together

This next section is about putting it all together. Whilst spending time with your baby, pay attention to their eye contact, where they look, where they point, and what they touch. If your baby is interested in something, get their attention to show them that you are interested too and that you want to speak or sign about it. This will also encourage them to do the same with you and will make it easier to be in joint attention with your baby.

When talking or signing about an object, first make eye contact by calling their name, tapping and/or waving so your child knows that something will happen. Then point to it, making sure your baby's eyes follow your finger. Move your finger to the object making sure your baby's eyes follow. Then look to the object as well. Once your baby's gaze returns to you, you can then sign or say the word. This way they will know what you are talking or signing about and can then link the sign or word to the object. If you are signing, sign slowly and clearly.

When talking or signing to your baby, repetition is always good as sometimes your baby may miss what you have said or signed.
[Video Clip 7]

[Video Clip 8]

Repeating a word or sign can help make sure your child has seen it and heard it, depending on their hearing status and use of hearing technologies. This will help them to learn and to develop their language skills.

When putting it all together and trying out these tips, remember that you don't just have to do them whilst playing with your baby. There are many great opportunities to engage and communicate with your child. For example, dinnertime, bath-time, and nappy changing. Changing your baby's nappy is a really great time to engage with your baby as they will be focussed on you. Here you have an opportunity to maintain eye contact and be animated in your facial expressions, and can also talk or sign to keep your child's attention. You can also try the tips in this video whist reading a book with your baby, making sure you are sitting in a position where your baby can easily see the book, and your face and hands.

The videos we have just seen are all about ways to make it easier to communicate with your baby and to encourage their communication. These were:

1. Getting down to eye level so it is easy for your baby to see you, make eye contact, and hear you if they are using hearing technologies.

2. Notice what your baby is focussing on, join in with animated facial expressions, and talk or sign about what they are interested in. To let your child know you are interested and want to tell them something, make sure you get their attention.

3. When you want to talk or sign about what your baby is interested in, use attention getters such as calling their name near and clear (particularly if your child is using hearing technologies), tapping, waving close to your baby's face, moving your face into their line of vision, and placing objects or signs in their field of vision. Be persistent and they will be more likely to look to you.

4. When they do look at you, respond quickly and with warm, animated facial expressions.

5. Keep a lookout for when your baby is trying to get your attention with eye contact and gestures.

6. Respond to these by joining in, showing your interest using facial expressions, and by talking or signing to them about what they are showing you or trying to tell you about.

7. When you are talking or signing to them, use a more child friendly voice and/or signing style.

8. Use lots of repetition when you talk or sign, and use facial expressions to support what you are saying or signing. Also, give your baby a little more time to process what you are saying and/or signing.

9. Remember that you can do all of these things in everyday situations with your baby.

This is what we would like you to try. Try using these tips in situations such as playing with your baby, reading a book, changing their nappy, on the bus, having a meal, or having a bath. We are running this study to see if using these tips makes communication easier.

So, what do you need to do?

We would like you to trying using these tips in a few everyday situations over the next two days. Try using them in any situation you and your baby are involved in. When the researcher comes back to visit, we would like you to try out these tips whilst we video record you and your baby playing together, like today's recorded play session. We are interested to see if you find this video helpful as we value your thoughts and feelings about the helpfulness of these tips.

Thank you for your interest in our study. We hope that both you and your baby will have fun taking part!
