# Supplementary material for: Assessing a Video-Based Intervention to Promote Parent Communication Strategies with a Deaf Infant: A Feasibility and Acceptability Study
Source: J Clin Med. 2022 Sep 7;11(18):5272. doi: 10.3390/jcm11185272 (PMC9505164; doi:10.3390/jcm11185272)
Supplement: Supplementary file 1 [file jcm-11-05272-s001.zip › Supplementary Materials S2_CodingManual.pdf]

Supplementary Materials S2: Coding Manual

Contents

Accessibility of Language Protocol 1
  Visual accessibility 1
  Aural accessibility 3
Semantically Contingent Talk Protocol 5
Repetition Protocol 6
Attention Getting Strategies Protocol 10
  Attention getting episodes 11
  Attention Getting Episode: Attention Getting Strategies Used 12
  Attention Getting Episode: Function 14
  Attention Getting Episode: Outcome 15
Infant Directed Sign Protocol 16

Accessibility of Language Protocol

This coding scheme was designed by the authors and developed using an iterative consensus approach with an independent coder, by piloting a subset of the total sample.

TIERS: Position (visual accessibility), Proximity (auditory accessibility), Offshot – code simultaneously

Visual accessibility

TIER: Position

In this tier, select which position the parent is in, in relation to their child, every time the parent makes and utterance. Parent utterances are marked in the *CDS* (Child Directed Speech) tier. Using the *Grid* tab on the right-hand-side select CDS. This will provide you with a list of every parent utterance. For every utterance, click on it and it will highlight the utterance on the *CDS* tier. Select on the tier below (the *Position* tier) the position of the parent. There are 4 positions the parent can be in: Eye Level + Face Very Accessible, Eye Level + Face Fairly Accessible, Not Eye Level but Face Fairly Accessible, Not Eye Level - Face Difficult to Access

To measure the visual accessibility of parental utterances, code whether the parent is at eye level with the child (or not) during utterances.

Eye level is considered to be:

- When the parent is in a position that allows their head to be level with their child's
- They can be roughly half a head above or below their child's head
- If the parent is a head above the child (mouth/chin above child's head), this is 'Not Eye Level but Face Fairly Accessible'
- They cannot be behind the child – even if heads are level, if they are behind the child this is not eye level.

<u>Four positions:</u>

1. Eye Level + Face Very Accessible
    - Parents are at eye level
    - Position examples:
        - Child is sitting on the floor and parent is lying on their stomach or side
        - Child is sitting on a chair and parent is sitting on the floor
        - Child is sitting on the floor and parent is sitting but has leaned forward to lower their head
        - Child is standing and parent is sitting on the floor
        - In some cases child is standing and parent is sitting on a chair leaning forward
    - Parents' faces are very easy for the child to access – meaning parents faces are in front of the child's or slightly above or to the side
    - It is very easy for the child to access their parent's face

- Minimal effort for child to see parent's face i.e., the child only needs to glance up/tilt head slightly up, glance to the side/turn head no more than 45° to the left or right

2. Eye Level + Face Fairly Accessible
   - Parents are at eye level
   - Position examples:
     - Child is sitting on the floor and parent is lying on their stomach or side
     - Child is sitting on a chair and parent is sitting on the floor
     - Child is sitting on the floor and parent is sitting but has leaned forward to lower their head
     - Child is standing and parent is sitting on the floor
     - In some cases child is standing and parent is sitting on a chair leaning forward
   - Parents' faces are fairly easy for the child to access – meaning parents' faces are not quite in front of the child's
   - It is fairly easy for the child to access their parent's face
   - Some effort for child to see parent's face i.e., they need to turn their head to the side more than 45°, turn head fully to the left or right = head turned 90° (child shouldn't need to fully look up as parents are at eye level, but can include when child only needs to glance up/tilt head slightly up as parent could be half a head above or below child's head, which is still eye level)

3. Not Eye Level but Face Fairly Accessible
   - Parents are not at eye level but are almost at eye level
   - Position examples:
     - Child is sitting on the floor and parent is sitting on the floor face-to-face (parent is roughly a head higher – mouth/child above child's head)
     - Child is sitting on the floor and parent is sitting on the floor slightly to the side of the child (parent is roughly a head higher – mouth/child above child's head)
     - If child needs to twist body as well as turn head this is 'Not Eye Level - Face Difficult to Access'
   - It is fairly easy for the child to access their parent's face
   - Some effort for child to see parent's face i.e., child needs to look up/tilt their head up fully, turn head to the side more than 45°, turn head fully to the left or right = head turned 90°, child needs to look up and to the side

4. Not Eye Level - Face Difficult to Access
   - Parents are not at eye level and it is difficult for the child to see their parent's face
   - Position examples:

        - Child is sitting on parent's lap, both facing forwards e.g., typical book reading position, and child needs to twist round to see parent's face/head turn more than 90°
        - Parent is behind child (even slightly), meaning child needs to twist body as well as turn head – even if twisting slightly i.e., any more than a 90° head turn
        - Parent is standing up and child is sitting/standing on the floor
    - It is difficult/very difficult for child to access parent's face
    - Effortful for child to see parent's face i.e., child needs to strain upwards, turn around, twist body round with head turn, more than 90° head turn needed

NB. If parent or child leaves the room, the play session has paused therefore, no coding happens in this time.

Auditory accessibility

TIER: Proximity

In this tier, select what proximity the parent is, in relation to their child, every time the parent makes an utterance. For every utterance, click on it and it will highlight the utterance on the *CDS* tier. Select the proximity of the parent in the *proximity* tier. There are 3 levels of proximity the parent can be, in relation to their child: Very Close, Close, Not Close, Far.

1. Very Close
    - Parent is very close to child
    - They are within the child's space i.e., within 1 metre of the child AND their head is very close to their child's head
    - Position examples:
        - Parent's face is touching child's face
        - Parent's face is very close to child's face (child could touch parent's face)
        - Parent's head is behind but very close to child's head e.g., child is sitting on parent's lap (both facing forwards)

2. Close
    - Parent is close to child
    - They are within the child's space i.e., roughly within 1 metre of the child around arm's length from child
    - Position examples:
        - Parent and child sitting face to face
        - Parent sitting parallel/next to child
        - Parent sitting behind child

3. Not close
    - Parent is not close to child

- They are outside of child's space – over 1 metre away (more than arm's length away) but no more than 2 metres away
- Position examples:
    - Parent is sitting/lying and child has crawled/walked away a couple of metres or so, meaning parent no longer within child's space
    - Parent is standing but in child's space

4. Far
    - Parent is far from child
    - They are roughly more than 2 metres away from child
    - Position examples:
        - Parent is at opposite end of room to child
        - Parent is standing outside of child's space

TIER: Offshot

Both tiers (*Position* tier and *Proximity* tier) have an option in the dropdown for offshot. Select offshot if the child and/or parent are not in shot of either cameras (and you cannot tell the position and/or proximity).

Semantically Contingent Talk Protocol

The coding scheme reported in McGillion et al. [65] was followed with minor adaptations. Adaptations were necessary to account for other aspects of the intervention for example, repetition. In cases where the parent repeated their contingent utterance several times, the time window restriction in which an utterance could be coded as contingent was waived. This was only the case if the child was no longer looking at the referent but was also not focussing on something new (e.g., the child was looking at their parent).

All caregiver utterances were coded for semantic contingency on the infant's focus of attention in the 5-second window preceding the utterance onset [63,64].

Utterances were coded as:

1. Contingent: if they referred to an object the infant was holding and/or looking at, or had referenced by a point, give or show gesture, or if they were related to an activity the infant was engaged in

2. Non-contingent: if they referred to an object or activity that the child had not attended to within the 5-second time window

3. Other: if they were not specifically contingent nor non-contingent (e.g., place filling expressions such as 'good girl', imperatives, saying the child's name in isolation etc.)

4. Imitation: if they were imitations of any non-lexical sounds made by the infant in the preceding 5 seconds

5. Inaudible: if the majority of the utterance could not be transcribed

Repetition Protocol

This coding scheme was based on similar studies [66,67,68]. Repetitions must occur within three utterances following the source utterance to take into account the limitations of short-term memory [66,68], with an additional restriction that they had to occur within a time window of 5 seconds. This time limit was chosen based on the literature that suggests a latency window within the range of 2 to 7 seconds between events, to allow for detection of links (i.e., the perception of temporal relations between the occurrence of events), given the infant's limited information processing capacities [69].

TIER: Repetition

- Go through the child directed speech (CDS) tier and look for occurrences of repetition in any form
- Code every occurrence of caregiver repetition in the *Repetition* tier (a dependent tier of the CDS tier)
- Only code occurrences of utterance repetition and not repetition within an utterance e.g., 'up, up, up'
- Code all lexical repetitions (including lexical exclamations) except for variations of 'oh'
- Only code non-lexical repetitions if they are sound effects i.e., eating/drinking noises, kissing noises (not actual kisses but the sound effect of a kiss e.g., 'kiss the teddy, mwah!'), animal noises (lexical and non-lexical), tickling noises, and vehicle sound effects
- Code imperatives i.e., 'look'
- Code place fillers i.e., 'well done', good girl' etc.
- Do not code the repeated use of the child's name as this is an attention getter and coded separately

When making a repetition annotation you will have a choice of 5 types of repetition to select from: *Exact Repetition, Exact+Expansion, Partial Repetition, Partial+Expansion, and Reframing*

- Any type of repetition of a preceding utterance **MUST be within three utterances from the original, unless it is more than 5 seconds from the original utterance**

- Exception to the 5 second/3 utterance rule = if there is an attention getting episode in between the two utterances

NOTE

- If an attention getting episode occurs in between repeated utterances, waive the 5s rule. For example, 'it's yellow', AG episode: 'childsname', tap, tap, wave, 'Childsname', 'it's yellow'

- Watch out for responses to infant communicative acts. For example, if parent says, ‘it’s there’, ‘it’s over there’ whilst referring to a ball by pointing. The child then points to the ball and parent responds by saying, ‘yes, it’s over there’. This last parent utterance is not a repetition
- If a repetition occurs with the same action, code as repetition. If it occurs with a different action, do not code as repetition
- Don’t code repetition when parent is singing a song or saying a nursery rhyme

A description of how to identify each type of repetition is described below:

- Exact Repetition
  - When an utterance is an exact (i.e., verbatim) repetition of the source utterance
  - Exception: addition/deletion of ‘childsname’, gasps, “words” with an ‘&’ prefix (i.e., nonsense forms and sounds), and variations of the words ‘yes’ and ‘no’ in the repeated utterance

- Exact+Expansion
  - When an utterance is an exact repetition of the source utterance but expanded upon with additional information
  - Note: additional information can be at the start of the utterance
  - For example:
    - ‘are you singing?’ ‘are you singing Twinkle Twinkle?’
    - ‘Go’, ‘go again’

- Partial Repetition
  - Parts of the source utterance are repeated i.e., one or more major units within an utterance (e.g., repetition of the subject phrase, or repetition of the semantically relevant part such as ‘where are the keys?’, ‘the keys?’) with addition and/or deletion of other words/sounds
  - Repetition of an utterance with a semantically similar substituted minor unit e.g., ‘it’s a cup’, ‘that’s a cup’, or ‘fill it up’, ‘fill her up’ or, ‘who is this?’, ‘who is it?’
  - When an entire utterance is repeated but without a verb
  - Changes in word order i.e., repetitions that repeat all words from the source utterance but in a different order (e.g., ‘the tower goes up’, ‘up the tower goes’)
  - Varied repetition of the phonological sound features e.g., ‘dog’, ‘doggie’
  - Examples:
    - ‘Shout monkey’, ‘you shout him’
    - ‘Come see monkey’, ‘come and see him’

- Partial+Expansion
  - When part of the previous utterance is repeated and expanded upon with additional information
  - Note: additional information can be at the start of the utterance
  - For example:

- ‘Is it lights?’, ‘lights on the front of the car?’
- ‘That’s the house’, ‘the farm house’
- ‘Can you hear it?’, ‘can you hear the tractor?’ (but the reverse i.e., ‘can you here the tractor?’, ‘can you hear it?’ = Partial Repetition) or ‘close it’, ‘close the lid’ or ‘can I have the banana?’, ‘can Mummy have the banana?’

- Reframing
  - When the previous utterance is reframed or paraphrased i.e., repetition of semantic content rather than grammatical units – the utterance is semantically the same but said in a different way. Only code as ‘Reframing’ if the utterance is a true paraphrase and does not qualify as a complete or partial repetition
  - For example:
    - ‘Look what else is in there’, ‘look in the bag’
    - ‘where’s the raisins gone?’, ‘where did they go?’
    - ‘Look’, ‘are you watching?’
    - ‘Do you want to open your tepee with the key?’, ‘are you gonna unlock your tepee?’
    - ‘Is that nice?’, ‘that delicious?’ – i.e., changing the semantically relevant part to something semantically similar. (‘you’ substituted with childsname’ and vice versa when the rest is a partial or exact repetition of the source utterance is not reframing)
  - Examples from Snow [66]:
    - "Pick up the red one. Find the red one. Not the green one. I want the red one. Can you find the red one?"
    - "Give mummy all the red toys. I would like all the things that look like this. Can you give me all the red things?"

Things to watch out for

- Sometimes it might seem like a repetition has occurred but that might not be the case. For example:
  - Child gives parent a toy and parent says, ‘thank you’. Child then gives parent another toy and parent says, ‘thank you’ again. This is not repetition as the parent is simply reusing the same phrase for the same action. If the parent says ‘thank you’ twice after receiving a toy, this is repetition
  - Similarly, parent stacks a brick and says, ‘that’s a brick’, then stacks a second brick and says, ‘that’s another brick’. This is not repetition
  - Similarly, parent says ‘look’ to look at one thing then says ‘look’ again but referring to something else. This is not repetition
  - A parent might have false started/stumbled resulting in some repetition e.g., ‘ooh, we've got, we've got Grandad’. This is not a repetition
  - Responding to child e.g., child makes sound and mum says ‘yeah!’ child makes sound again and mum says ‘yeah!’ again – this is not repetition as responding to child

- Responding to environmental sounds e.g., toy makes a 'beep-beep' sound and parent says 'beep-beep', toy repeats sound and parent repeats 'beep-beep'
- Some games e.g., saying 'Mr crab is coming to get your nose', got your nose' with actions and repeating this as a sort of game, is not repetition. Repeating the same sound/word/phrase and action is usually repetition e.g., snake goes 'ssss' with action, 'ssss' with action, 'ssss' with action. However, it is not repetition if repeating as part of a game. Further examples include, 'hide the ball', 'where's the ball', 'there it is', 'hide the ball', 'where's the ball', 'there it is'. Or, 'does he go in here?', 'no!' 'does he go in here?', 'no!' etc. when trying to fit a jigsaw piece in different holes.
- If parent repeats what the child says (this is rare), do not code as repetition

Attention Getting Strategies Protocol

This coding scheme was designed by the authors and partially based on similar studies [22,54]. The coding scheme was developed using an iterative consensus approach with an independent coder, by piloting a subset of the total sample.

TIERS:
Attention Getting Episode, Attention Getting Strategies, Function, Outcome

You will need to make annotations in all of these tiers.
DO NOT CODE WHEN PARENT IS OFFSHOT.
How to annotate in each tier is described below in detail.

1. Look out for any instance where the parent is attempting to change their child's visual attention by directing their child's visual attention to either:
   - Themselves (i.e. to their face)
   - Their signs
   - An object/part of an object
   - An event

2. Attempts to direct child's visual attention will involve the use of attention getters

   - An attention getter to direct child's visual attention = the use of a signal to elicit attention. Signals to elicit attention can be:
     - Calling child's name
     - Another vocal cue e.g., hello/hey/saying a pet-name
     - Touch e.g., tapping, caressing, poking, stroking
     - Waving in their child's line of vision
     - Moving their face/head into child's line of vision
     - Sign displacement = producing/moving a sign(s) into child's line of vision
     - Use of vibration/loud noise e.g., banging the floor
     - Moving an object to their face (when speaking or about to speak i.e., trying to direct child's gaze to face using object)
     - An imperative to direct child's visual attention (i.e., instructing the child's visual attention e.g., look, look at me, look at that, look over there, watch, watch me etc.)
     - **These signals are referred to as attention getters or attention getting methods**

Common problems:

- **Moving face/head into line of vision can be difficult to distinguish from the parent getting down to eye level**
  - If the parent moves down into line of vision and settles there i.e., shifting position – this is parent getting down to eye level
  - If the parent moves face/head into child's line of vision and soon after retracts, or follows child's moving line of vision, this is an attention getter. Moving

into line of vision is often accompanied by other attention getters e.g., calling child's name

- **Sign displacement**
    - Sign moved into child's line of sight = attention getter (to language)
    - Sign moved near object FOA = accommodative strategy and not an attention getter
    - Guiding child to make sign = accommodative strategy and not an attention getter

3. See next section for details on how to code in each tier

Attention getting episodes

TIER: Attention Getting Episode

- **Identifying an Attention Getting Episode:**
    - Mark all the time the parent attempts to elicit their child's attention i.e., when the parent uses an attention getter in isolation, a combination of attention getters or a combination of attention getters and imperatives (only the visual attention directing ones). DO NOT code imperatives in isolation or a combination of imperatives unless combined with an attention getter(s).
    - Include parent gesture or object manipulation in the episode but only if it occurs WITH an imperative (e.g., 'childsname', 'look' and holds a ball in front of child)
    - An attention getting episode can be the occurrence of one method in isolation OR a combination of methods to elicit child's attention

- **The beginning of the Attention Getting Episode** is the moment the parent engages with the child using the first (or only) attention getting method
    - If the first (or only) method is calling the child's name, this will already be coded in the CDS tier, so you can use the beginning of that annotation to mark the beginning of the episode
    - If the first (or only) method is another vocal cue or an imperative, this will already be coded in the CDS tier so you can use the beginning of that annotation to mark the beginning of the episode
    - If the first (or only) method is tapping (or any type of touch), the beginning is the first frame that you judge the parent has made contact with the child
    - If the first (or only) method is waving in the child's line of vision, the beginning is the first frame that you judge any part of the parent's hand to be in the child's general field of vision (including periphery)
    - If the first (or only) method is moving into the child's line of vision, the beginning is the first frame that you judge the parents face/any part of head to be in the child's general field of vision (including periphery)
    - If the first (or only) method is moving/producing a sign(s) in the child's line of vision, the beginning is the first frame that you judge the parent's hand(s) to be in the child's general field of vision (including periphery)

- **The end of the Attention Getting Episode is** the moment the parent stops engaging with their child using an attention getting method. This will usually happen if the child responds or the parent gives up. The parent might pause to wait for their child to respond and if they don't, the parent might continue. This is still one episode as the parent is still persisting.
  - If the last (or only) method used is vocal, the end is the last frame that you judge the parent vocalised. In some cases the CDS tier can help you to identify this frame
  - If the last (or only) method used is touch, the end is the last frame that you judge the parent has made contact with the child
  - If the last (or only) method used is waving in the child's line of vision, the end is the last frame that you judge any part of the parent's hand to be in the child's general field of vision (including periphery)
  - If the last (or only) method used is moving into the child's line of vision, the end is the first frame that you judge the parents face/any part of head to be in the child's general field of vision (including periphery)
  - If the last (or only) method used is moving/producing a sign(s) in the child's line of vision, the end is the first frame that you judge the parent's hand(s) to be in the child's general field of vision (including periphery)
  - **Common Problem: determining end of episode – has it ended and another begun or is it one big attention getting episode?**
    - If the episode ends with success, any attention getters afterwards will be the beginning of another episode
    - An indication the parent has given up/ended the episode will be that the outcome was unsuccessful and the parent no longer continued to get attention before speaking/signing. If the parent attempts to get attention, stops and it is unsuccessful, goes on to talk about FOA and then starts attention getting again; this is 2 episodes. The parent gave up as she continued to speak without child's attention but then decided to try again, starting a new attention getting episode

- Once you have identified all attempts to direct the child's visual attention on the *Attention Getting Episode* tier, you will need to code the Method, Function, and Outcome

- The *Attention Getting Episode* tier has dependant tiers for Method, Function, and Outcome

Attention Getting Episode: Attention Getting Strategies Used

TIER: A.G.E Method

Navigate through each attention getting episode you have coded using the Grid tab to code in this tier.

- In this tier, descriptively type the method or methods that were used in order of occurrence using the key below:

Method	Code
Calling child’s name	Name
Another vocal cue or imperative	Type verbatim what was said
Touch	Type of touch e.g., tapping, poking etc.
Waving in child’s line of vision	WLOV
Moving into child’s line of vision	HLOV
Sign displacement in child’s line of vision	SLOV
Use of vibration/loud noise e.g., banging floor	Type action used e.g., ‘bang toy on floor’
Moving object to face	Obj face
Clapping	Clap
Clapping in child’s line of vision	Clap LOV
Moving child into parent’s LOV	Move child into LOV (be careful this isn’t to make eye level easier/possible

- Example method: Name, tapping, HLOV, look, look at Mummy
- Example method: HLOV, name, name, WLOV, name

Note: All uses of child’s name are not attention getters e.g., ‘here you go, Childsname’

Note: LOV means in visual field (right in front of face or to side of face/periphery) not just generally in front of/near child

When parent is using an attention getter(s) to get their child’s attention to an object, also include if the parent manipulated the toy in some way such as moving, shaking it, tapping the object, or pointing to the object, using below codes:

Obj man	Parent manipulating toy by shaking it, moving it, holding it out etc.
Obj tap	Parent tapping on toy
Obj point	Parent pointing to object/parts of object
Obj LOV	Parent puts toy in child’s line of vision

Attention Getting Episode: Function

TIER: A.G.E Function

Navigate through each attention getting episode you have coded using the Grid tab to code in this tier.

- In this tier, select the function of the attention getter i.e., what was the aim of the attention getter? Where did the parent want the child to look? You will need to select from 1 of 5 options. These are:

**DVA to Self**	Goal for child to attend to parent's face or signs (i.e., language input)
**DVA to Obj FOA**	Goal for child to attend to object that is child's FOA (see FOA definition below)
**DVA to Obj Non-FOA**	Goal for child to attend to object that is NOT child's FOA (see FOA definition below)
**DVA to Obj Initiate**	Goal for child to attend to object when child has no FOA/is not doing anything, to initiate joint attention
**Unclear**	Parent's motives unclear. If a failed attention getter occurs in isolation, code as unclear

- If parent is object manipulating and says, what's this, what's mummy got? This is not an attention getter. Parent talking about object but there is no attention getter to get child's attention to talk about object. Manipulating the object could be a method to direct attention to it but this isn't coded unless parent says and attention directing imperative.

- **FOA definition**

FOA = infant focus of attention. An object can be considered the infant's FOA if the child attended to it *at any point* in the ***5 seconds preceding the A.G.E's onset***.

Markers of infant attention

- When an object (or action on an object) is the focus of the infant's attention e.g., building a tower with stacking cups, pushing a car along the floor etc. Behaviours that help identify the infant's focus of attention include:
  - looking at the object (this may be fleeting)
  - touching/manipulating the object (manually or orally)
  - showing/giving the object to caregiver
  - pointing (index or open hand) to the object
  - reaching for an object
  - pre-linguistic vocalisations that co-occur with any of the above behaviours
  - naming the object (rare in 12m olds, occasional in 18m olds)
- Pre-linguistic vocalisations alone are not sufficient to indicate FOA and can only be considered as evidence of the infant's FOA when appearing in conjunction with another indicative behaviour, EXCEPT in the rare situation that the infant is reliably naming the object

- Touching or holding an object alone is not enough to indicate attention i.e., idly holding the object and looking elsewhere. They need to be looking at it or giving/showing it to parent
- Looking at/showing/giving/pointing in isolation can be considered enough to indicate focus of attention

Attention Getting Episode: Outcome

Navigate through each attention getting episode you have coded using the Grid tab to code in this tier.

TIER: A.G.E Outcome

- In this tier, select the outcome of the attention getter i.e., did the child look to target? Did the child look elsewhere? Or did the attention getting episode fail to elicit/direct child's attention? You will need to select from 1 of 4 options. These are:

**Successful**	Child looks to intended target (the function)*
**Unsuccessful**	The child does not change attentional state/respond
**Offshot**	Child is offshot/child's gaze is unavailable to determine outcome
**Ambiguous**	If child responds to an unclear attention getter

*Caveat: if the method is moving object to face and child looks to object next to face or in front of face, code as successful as parent's face is in child's visual field

- If the function of the attention getter is unclear:
  - Code as unsuccessful if the child does not change attentional state/respond
  - Code as ambiguous if the child changes attentional state/responds

TIER: A.G.E Notes

- This is a free text tier. You can use it for additional comments and observations about each Attention Getting Episode e.g., parent is trying to get child's attention and moves child to face her so she can use the HLOV method more easily. You could note this here
- If you feel there are any cases that are borderline i.e., the attention getting episode might not have been one, type 'BL'
- If you feel one of the methods used might not have been a method, type 'BL' and whatever the method was that you were unsure of e.g., HLOV BL

Infant Directed Sign Protocol

This coding scheme was based on definitions from Waxman & Spencer [22].

TIERS: SSE, Accommodative strategies – code simultaneously

TIER: SSE

In this tier, mark every instance where the parent uses sign supported English (SSE).

- An instance of SSE can be a standalone sign produced simultaneously alongside the corresponding spoken word (e.g., signing *blue* when saying *blue* in the utterance, "that's a blue ball")
- An instance of SSE can be a string of signs produced simultaneously alongside the corresponding spoken utterance (e.g., signing *where lion* when saying, "where is the lion?")

TIER: Accommodative strategies

- Navigate through each instance of SSE you have coded using the Grid tab to code in this tier
- For every instance of SSE, determine if any accommodative strategies were used – this is when signs are adapted to accommodate infant visual attention
- There are 3 accommodative strategies to code for:

    1. Sign displacement: if the parent signs outside of the signing space to make it easier for the infant to see the sign(s). For example, signing next to the referent such as a toy or picture in a book

    2. Guiding child to make the sign: if the parent helps or guides the child to make a sign by manipulating the child's hands

    3. Sign in child's line of vision: if the parent signs/moves a sign into the child's line of vision

- If an accommodative strategy is used, select which strategy in this tier
