# Supplementary material for: Assessing a Video-Based Intervention to Promote Parent Communication Strategies with a Deaf Infant: A Feasibility and Acceptability Study
Source: J Clin Med. 2022 Sep 7;11(18):5272. doi: 10.3390/jcm11185272 (PMC9505164; doi:10.3390/jcm11185272)
Supplement: Supplementary file 1 [file jcm-11-05272-s001.zip › Supplementary Materials S3_InterventionDiary.pdf]

Participant number: ________________________________________

# Supplementary Materials S3: Intervention Diary

**Please record each time you try advice from the video**

Day 1 date: _______________

| Activity (playtime/lunchtime/book reading/general etc.) | For how long? | If you like, please add any comments/thoughts about trying the tips |
|---|---|---|
| | | |
| | | |
| | | |
| | | |
| | | |
| | | |
| | | |

Participant number: ________________________________________

**Please record each time you try the advice from the video**

Day 2 date: _______________

| Activity (playtime/lunchtime/book reading/general etc.) | For how long? | If you like, please add any comments/thoughts about trying the tips |
|---|---|---|
| | | |
| | | |
| | | |
| | | |
| | | |
| | | |
| | | |
