# Supplementary material for: Assessing a Video-Based Intervention to Promote Parent Communication Strategies with a Deaf Infant: A Feasibility and Acceptability Study
Source: J Clin Med. 2022 Sep 7;11(18):5272. doi: 10.3390/jcm11185272 (PMC9505164; doi:10.3390/jcm11185272)

Supplementary Materials S4: Participant-level Raincloud Plots Showing Changes from Pre- to Post-intervention for Each Behavior

Figure S1: Participant-level raincloud plots showing changes from pre- to post-intervention for visual accessibility of language

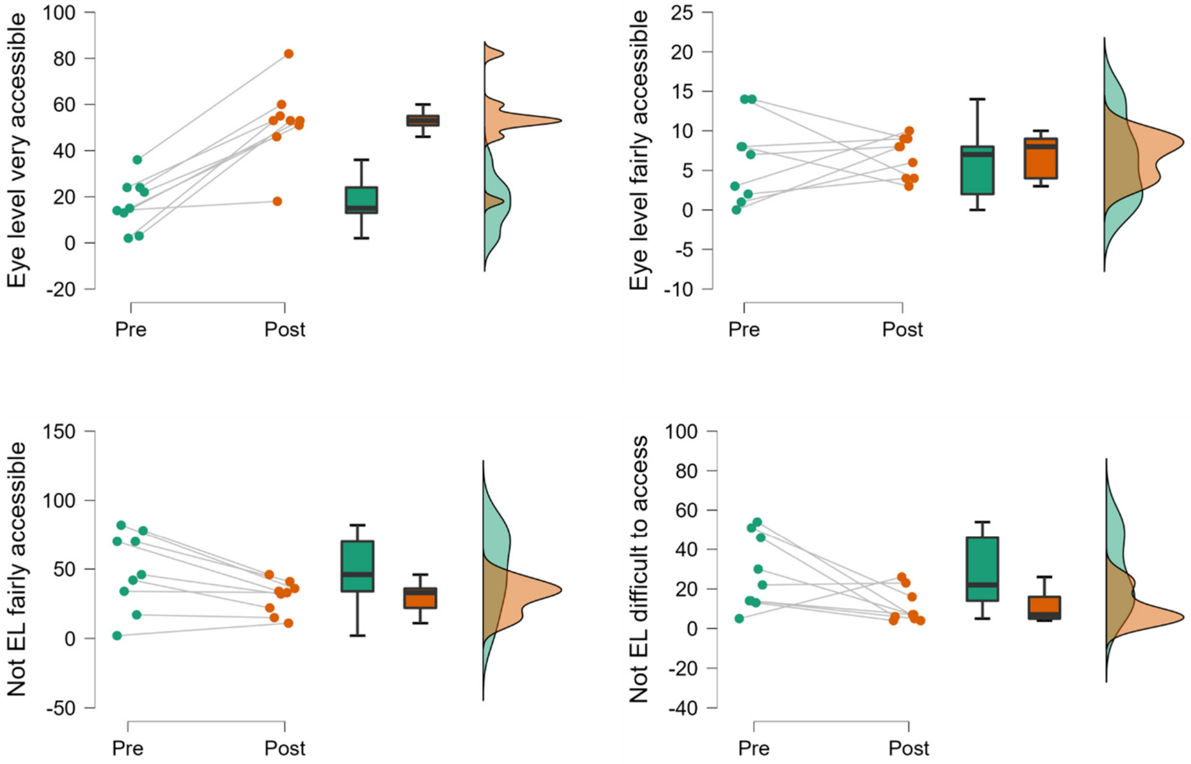

Note. EL = Eye level

Figure S2: Participant-level raincloud plots showing changes from pre- to post-intervention for auditory accessibility of language

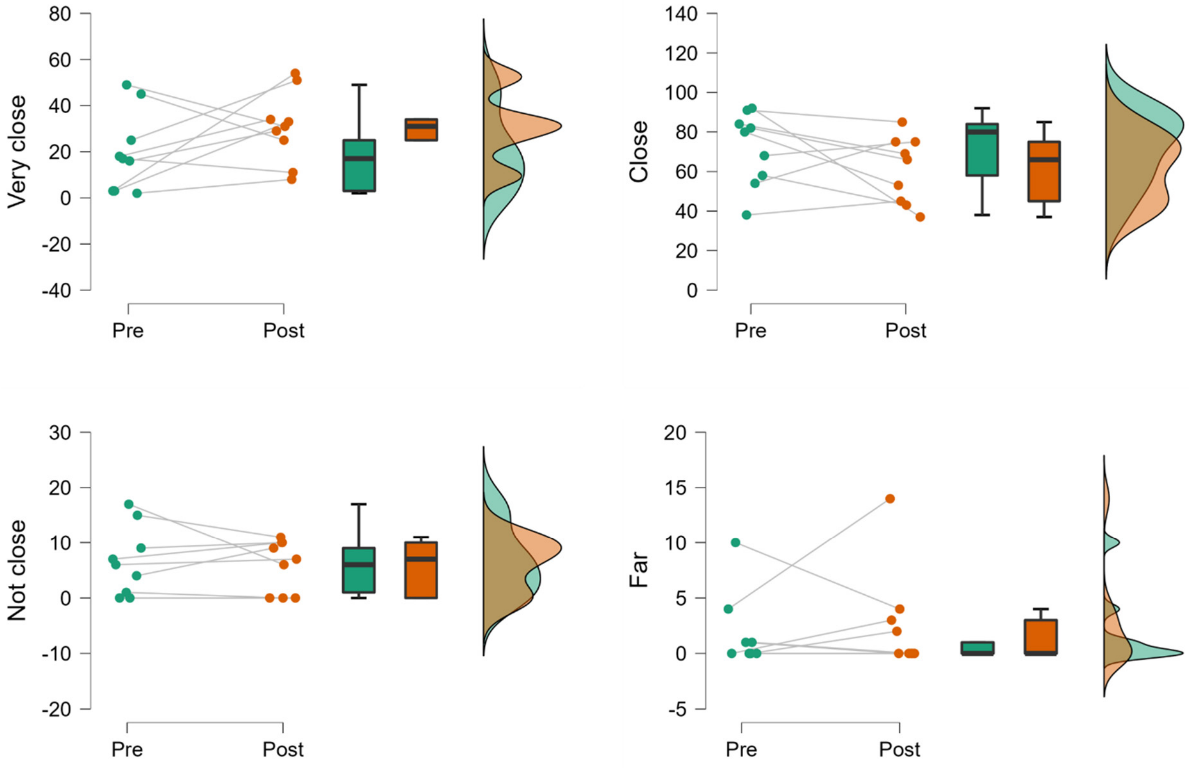

Figure S3: Participant-level raincloud plots showing changes from pre- to post-intervention for semantically contingent talk

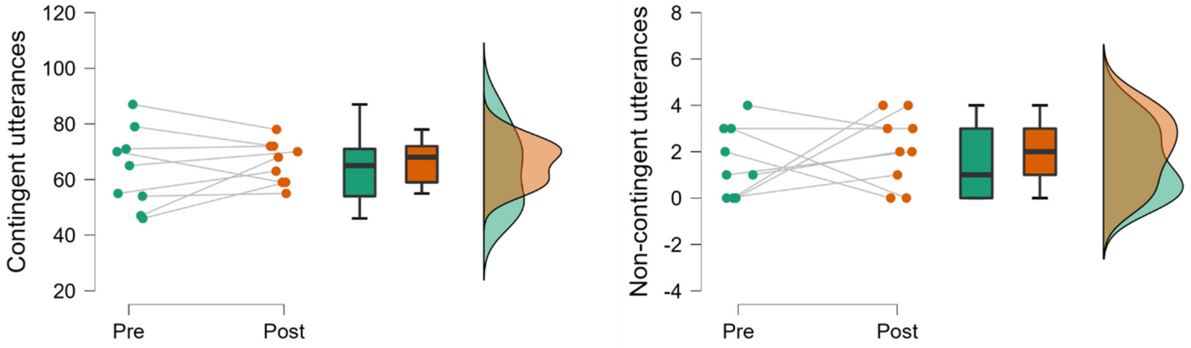

Figure S4: Participant-level raincloud plots showing changes from pre- to post-intervention for repetition in child directed speech

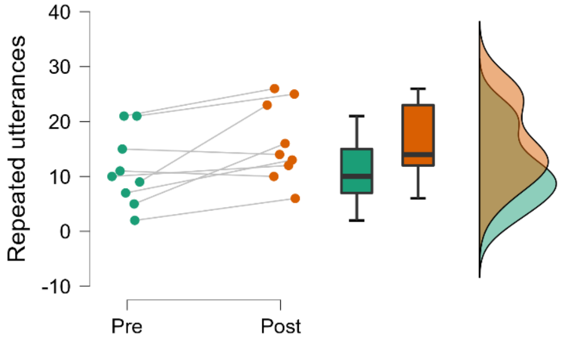

Figure S5: Participant-level raincloud plots showing changes from pre- to post-intervention for attention getting episodes and individual attention getting strategies

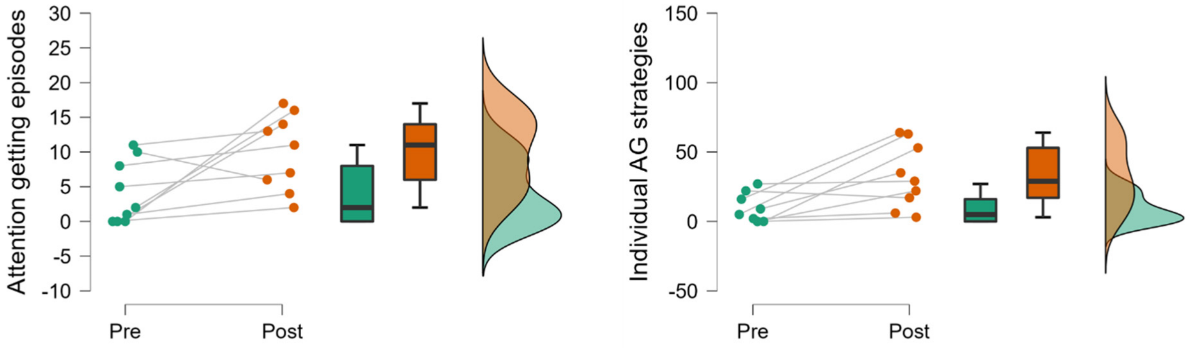

Note. AG = attention getting

Figure S6: Participant-level raincloud plots showing changes from pre- to post-intervention for function of attention getting episodes

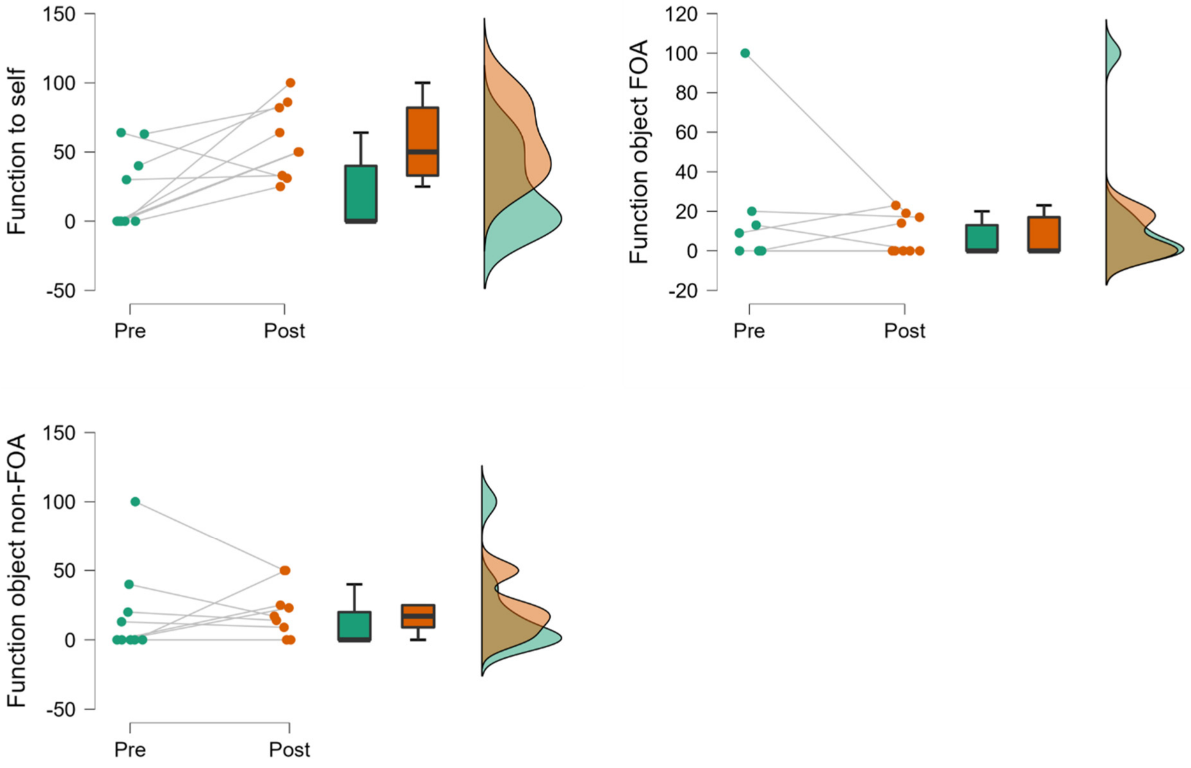

Note. FOA = focus of attention

Figure S7: Participant-level raincloud plots showing changes from pre- to post-intervention for outcome of attention getting episodes to elicit infant visual attention to self

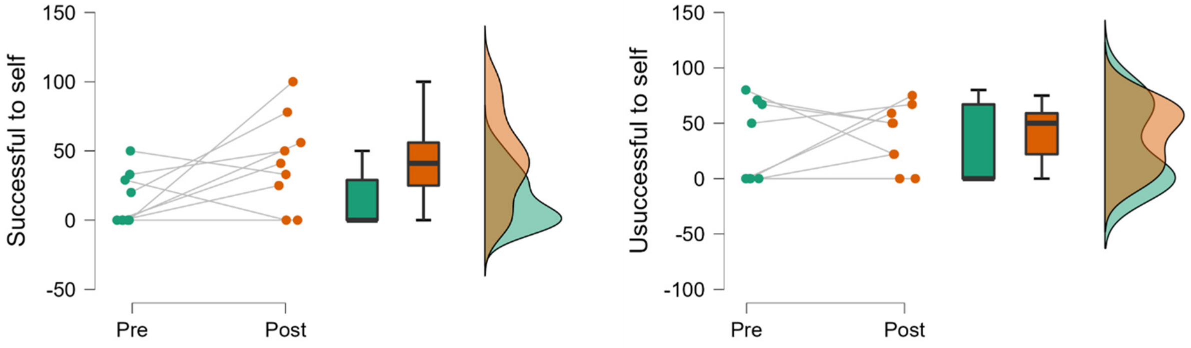

Figure S8: Participant-level raincloud plots showing changes from pre- to post-intervention for persistence

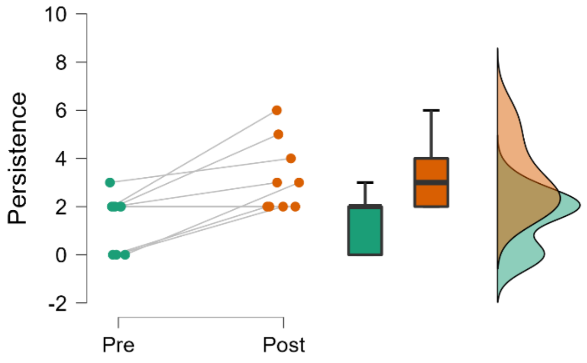

Supplement: Supplementary file 1 [file jcm-11-05272-s001.zip › Supplementary Materials S4_RainCloudPlots.pdf]
