# Supplementary material for: Assessing a Video-Based Intervention to Promote Parent Communication Strategies with a Deaf Infant: A Feasibility and Acceptability Study
Source: J Clin Med. 2022 Sep 7;11(18):5272. doi: 10.3390/jcm11185272 (PMC9505164; doi:10.3390/jcm11185272)
Supplement: Supplementary file 1 [file jcm-11-05272-s001.zip › Supplementary Materials S5_Study2VideoScripts.pdf]

Supplementary Materials S5: Study 2 Video Scripts

Contents

Link to video series 1

Introduction video: An Introduction to Supporting Early Communication Development 1

Video 1: How do Babies Learn to Communicate? 3

Video 2: Supporting the Communication Development of Deaf Babies and Toddlers 5

Video 3: Tuning-in and Responding to Your Baby’s Communication 7

Video 4: Supporting Access to Language 10

Video 5: Thinking About How We Communicate 15

Link to video series

https://www.ndcs.org.uk/information-and-support/language-and-communication/supporting-your-childs-learning/developing-language-and-communication-in-0-2s/

Introduction video: An Introduction to Supporting Early Communication Development

Welcome to this series, which provides information and tips on supporting the communication development of deaf babies and toddlers, with any level of hearing loss – from mild to profound. But before we get started, let's first think about your family's communication route.

You might be faced with lots of information about different communication approaches like sign language (for example, British Sign Language), or sign supported English, or auditory-oral approaches, which focus specifically on listening and spoken communication. And you might also be thinking about whether your child will use spoken language, sign language, or be bilingual in both.

When deciding between all the options many parents ask, 'what is the best approach to support my child's communication development?'. Research on this shows that choices are very individual and there is no clear evidence that one approach works best for *all* babies, as all families and children are different and have different circumstances. So the "best" choice is the one that suits your child and family.

You'll probably have met with your local Teacher of the Deaf, and may have met with other professionals too. Talking to the professionals working with your family can really help with your decision. It can also help to meet other families and people who have knowledge and experience.

The key is to make well informed choices for your family based on the information that is out there and what you feel best meets your needs. And remember, whatever approach or mix of approaches you choose, you don't have to stick with it permanently. If it doesn't feel right, or no longer works for your child and family, you can always adapt or change it.

No matter what stage you are at in your decision, the videos in this series provide information based on the best available research evidence, to help you support your child's development.

The videos fit with a range of different communication approaches. For example, whether you are using sign or focusing on listening and spoken communication, there are tips that fit with these options. And if you are using a mixture of both, there are tips on how to balance these. To decide on the tips that would work best with your family, it can help to talk to the professionals who are supporting you.

The first video in this series called, 'How do babies learn to communicate?' is a whirlwind tour through early development, looking at the communication skills that develop in the first 2 years. The rest of the videos are about what we can do to support this development for deaf babies with any level of hearing loss.

There's a lot of information and advice in these videos, so don't worry about taking it all in or getting the hang of it all straight away. It might be helpful to pause and make notes as you go along. And come back and watch parts again if you are trying out particular strategies.

When it comes to communication, learning is about doing. The more we communicate with babies, the more they learn how to communicate. Anything you can do to give them early learning opportunities will really help their development.

Video 1: How do Babies Learn to Communicate?

Right from the moment they are born, babies are learning, and one of the most important things they can learn about is how to communicate with other people.

Even though they can't use words at first, that doesn't stop babies learning about how people express themselves, and how to make themselves known to the people around them.

All they need for this learning, is interaction.

Babies love to look at you. When they do, they receive information about what other people are like and dedicated areas of the brain that process social information start to develop.

As we hold, soothe and interact with babies, we engage in a sort of conversation with them.

For example, we might reply to a hiccup and from 6 to 8 weeks, they might even smile back in response.

Responding to babies' noises, facial expressions, and movements begins the development of turn-taking, which is when one person does something and then the other responds.

Learning about turn-taking is an important part of learning to how to communicate.

When we communicate with babies, we naturally tend to adapt to their level. So at first, we reply to movements, facial expressions and sounds like hiccups and cooing.

Then, when they begin to babble and gesture like pointing, we respond more to these.

By adapting to their level when we communicate with babies, we actually help them to reach the next level of their development, whenever they are ready. This is how we support their development.

We all do this slightly differently, so every family finds their own rhythm and babies soon learn what to expect from their family routines.

At first, babies learn through face-to-face interaction. But as their eyesight develops they start to look to the outside world as well.

They even start to follow what we are looking at.

Then, from around 9 months, babies start to do something remarkable. They become able to enter into something called joint attention.

This happens when both you and your baby are paying attention to the same thing and both know you are doing so together.

This might not sound like much, but it is part of developing language, which is all about sharing attention to a topic of conversation.

Before they can start to talk, babies need to learn how to follow other people's attention, and how to direct it.

They often do this with babble and gestures like pointing. Sometimes, they will also look at you to check you've understood them.

When babies do babble and gesture, responding to them is incredibly rewarding and provides a great language learning opportunity for them.

By talking to babies about what they are interested in, we make it easier for them to learn how to talk.

Before we know it, babies start to say words themselves, and in time, can put words together into mini sentences.

Being able to communicate gives them the power to understand people, to make their needs known, and even to make friends.

Video 2: Supporting the Communication Development of Deaf Babies and Toddlers

Many of the developments that happen in a baby's first 2 years will be just the same for babies with hearing loss.

But there are some important aspects of learning to communicate that they will need help with.

When we interact with babies, we create lots of opportunities for them to learn about communication. For example, by responding to their sounds, facial expressions, and movements during face-to-face interaction, we help them learn about turn-taking.

When they are interested in something, we can make learning words and signs much easier by noticing what they are focussing on and talking or signing about it.

Sometimes, when babies are interested in something, they might babble or make a gesture like pointing.

Research suggests that responding to your baby's communication is incredibly powerful.

When you respond to them, babies learn that they can use sound and gestures like pointing as a way to share what they are interested in with other people.

Once babies have learnt that they ***can*** communicate with babble and gestures, they are then ready to start using words or signs to do this.

When we interact with babies with hearing loss, some of these opportunities to learn about communication can be missed.

For example, when you respond to your baby and communicate with them, they are more likely to miss that you are talking or signing to them, and can also find it harder to follow what you are saying or signing.

But you can play an important role in making sure that they have as many opportunities to learn as possible. So the videos in this series are all about ways we can do this. In the next video called, 'Tuning-in and Responding to Your Baby's Communication' we explore tuning-in to what your baby is interested in, talking to them about it, and responding to their attempts to communicate with you.

By actively trying to do this as much as possible, you can help increase your baby's language learning opportunities.

The 'Tuning-in and Responding to Your Baby's Communication' video, also looks at how to reduce the possibility that your baby may miss that you are talking or signing to them, by exploring ways to make it easier for your baby to see and hear you.

In the video called, 'Supporting Access to Language' we look at ways to make it easier for your baby to follow what you are saying or signing, so that it is easier for them to learn.

You can do this by encouraging your baby to look at you when you are talking or signing to them.

You can also do this to make it easier for your baby to pay attention to what you are saying using their hearing and listening.

In the last video called, ‘Thinking about *How* we Communicate’, we look at ways you can adapt *how* you talk or sign to your baby that helps keeps them engaged and makes learning words and signs easier.

These videos should be compatible with your chosen communication approach, but to find what best suits your family you may want to adapt the tips and advice in discussion with the professionals who are supporting you.

You don’t need to watch these videos all at once but it might be helpful to watch them in order as they help build towards the goal of supporting communication in everyday situations and providing lots of language learning opportunities for your baby.

When trying the tips and advice in these videos, you don’t have to get the hang of it all straight away. In time and with practice it will all come together.

Video 3: Tuning-in and Responding to Your Baby's Communication

Tuning-in and responding to your baby's communication is really important for supporting their language development.

So the first part of this video is all about why this is important and what we can do. The second part looks at ways to make tuning-in and responding easier when your baby has any level of hearing loss.

Part 1: Tuning-in and responding, what's it all about?

Babies under 18 months of age are more likely to learn language if it is about what they are interested in.

If you are talking or signing about something different to what your baby is focussing on, they will find it difficult to switch their attention, and understand what you are talking about.

So an important way to support your baby's language development is to talk or sign about what they are interested in.

This is often called, 'following their lead'.

To do this, take a moment to watch your baby and notice what they are engaged in or what has caught their attention. Once you have noticed what your baby is focusing on, join in and talk or sign to them about it.

The key here is to tune-in and then talk or sign about what they are interested in.

This is something you may already be doing, but actively making time for this will provide more opportunities for your baby to learn.

Babies will often let you know what they are interested in by trying to communicate with you through babbling and gestures like showing or giving you something or by pointing.

Research shows that if you respond to them and translate what they are trying to say into words, this will *really* help them to learn to talk or sign.

So try to be on the lookout for your baby's babble, gestures and eye contact.

This might not happen very often. So it is really important to look out for when they are trying to communicate with you, so you can respond and turn *those* moments into language learning opportunities for your baby.

When you respond, try using warm, animated facial expressions as this will not only make it more fun for your baby, it will also encourage them to communicate more with you. This will help create even more learning opportunities for your baby.

So the key here is to look out for your baby's attempts to communicate and then respond by *translating* what they may be trying to tell you, all whilst using warm and animated facial expressions.

Once they start to use words themselves it is helpful to expand on what they say when you respond to them. This is when you add words to what your baby has said.

For example, if your baby *says*, “Duck”, you could respond by saying, “Yes duck! The duckie goes, ‘quack quack!’”

In time, they will be able to *combine* words to say longer sentences.

Part 2: Tuning-in and responding to your baby’s communication when your baby has any level of hearing loss

When following a baby’s lead and responding to them, there are 2 steps to making your responses great opportunities for them to learn language.

<u>Step 1</u>: they need to *notice* you are talking to them.

Babies are more likely to notice we are talking to them, and are more likely to pay attention to what we are saying, when we speak in a more lively, exciting and child friendly way.

<u>Step 2</u>: they need to be able *access* what you are saying.

When babies are interested in something, and you are talking to them about it, they will pay attention to what you are saying mostly by listening to you, but also by sometimes looking at you as well. This is how they can access what you are saying.

For babies with hearing loss, step 1 and step 2 can be trickier as they are *more* likely to miss that you are trying to tell them something, and can find it harder to access what you are saying.

But *you* can help make step 1 and step 2 easier. So let’s think about *how* you can do this.

When you are following your baby’s lead, it can be really helpful to encourage them to look at you *before* you speak or sign about what they are interested in. This can help to let your baby know you are about to tell them something.

Encouraging them to look at you, can also help make it easier for your baby to access what you are saying or signing.

Sometimes, you can try waiting to see if they will look at you before you respond.

The key is to try to make sure they are looking at you just before you talk or sign to them about what they are interested in.

Some children look at their parents more often than others, so this can be tricky.

But giving them a big smile and talking about what they are interested in when they do look at you, will encourage them to look at you more and more over time.

Learning to look at you more will make it easier for your baby to notice that you are communicating with them, and access what you are saying or signing.

So, how can you encourage your baby to look at you and support their access to language? The next video in this series called ‘Supporting Access to Language’ provides lots of information about how to encourage your child to look at you, *and* how to support their access to what you are saying or signing.

With practice, you will build up the ability to take turns with your baby and have back-and-forth conversations.

Keeping this going can be difficult, but by making time to tune-in to what has caught your baby’s attention, talking to them about it, making it easier for them to notice you are talking or signing to them, *and* can easily access what you are saying or signing, *you* can really make a difference.

Video 4: Supporting Access to Language

Learning language is all about learning how people use words or signs and sentences. But to learn how words or signs and sentences work, babies first need to be able to access them.

We access what people are saying by hearing them but also by seeing their lip-patterns, which is how your mouth moves when you talk.

This helps you to speech-read. You may have heard of this as lip-reading.

We also access what people are saying by seeing their facial expressions and natural gestures, which are the way we move our hands and bodies when we talk.

All babies, with or without hearing loss can learn from these gestures.

For example, you might point at something while talking, which can help make clear what you are talking about.

Both facial expressions and natural gestures play an important role in helping us understand what people are saying, so being able to see these is important.

For babies with hearing loss, accessing what you are saying can be more difficult.
For many babies it will be harder for them to hear your words clearly.

It can also be harder for them to notice when you are communicating so they may miss your lip-patterns, facial expressions, and natural gestures, and signs if you are using them.

But there is a lot you can do to support them.
You can support their auditory access.

This is all about ways to make it easier for babies to use their hearing and listening to access what you are saying, but this will depend on your baby's hearing levels.

You can also support their visual access.

This is all about encouraging your child to look at you, and making it easier for them to see you, when you are communicating with them.

By looking at you, your baby can see your lip-patterns, facial expressions, natural gestures and signs if you are using them.

If you are being supported by professionals to use a particular communication approach, you may be using techniques which focus more on auditory cues, hearing and listening.

Or you may be using techniques to focus more on visual cues and visual learning.

But for everyday situations, the key is to think about how to provide the best possible access to language for your baby and to give them as many opportunities as possible to communicate.

The next section of this video will look at the different ways you can support auditory access and visual access to language.

The final section will look at how you can combine and balance these to provide the best possible access to language for *your* baby in everyday situations.

Supporting Auditory Access to Language

Number 1 – Hearing Technologies

It is important to try to make sure your baby is wearing their hearing devices as much as possible when they are awake.

This can sometimes be hard to do, as babies often want to take them out. Try to be persistent at keeping them in. The more you try, the more likely they will stay in.

The more they are in, the more your baby will have auditory access to what you are saying, which will help them develop their hearing pathway.

And remember, it is also important to regularly check that your baby's hearing devices are working correctly. If you are not sure how to do this, you can always ask your Teacher of the Deaf or Audiologist.

Supporting Auditory Access to Language

Number 2 – Be Near and Clear

For babies learning to listen with their hearing aids or cochlear implants, try to be within 2 metres of your baby when you speak to them, and try to speak clearly. This makes it easier for them to access your speech.

When your baby starts to crawl and walk, this will sometimes be harder to do.
But don't worry, you don't need to follow your baby around all the time.

If they are on the move and then settle somewhere further away from you, this is a good opportunity for you to move to be nearer to them when you want to talk to them.

When sitting with your baby, sitting next to them can also help them practice listening and develop their listening pathway.

You can also sit with your baby on your knee.

Supporting Auditory Access to Language

Number 3 – Managing Background Noise

Background noise can make it harder for your baby to use their hearing and listening to access what you are saying.

But you can create a good listening environment for your child.

You can do this by switching off the TV or radio if you are not actively watching or listening to them.

This will also make it easier for your baby to pay attention to what you are saying and take turns during the conversation with you.

Think about when you turn on noisy machines like the washing machine. At times when you are communicating lots with your baby like at mealtimes, it can be helpful to make sure the washing machine or dishwasher is off.

When your baby is playing with a musical or noisy toy, try to wait for quiet moments before speaking to your baby.

For times when background noise is hard to avoid or difficult to manage, radio aids can be really helpful. A radio aid is a piece of technology you can wear that can help your baby to hear you better in noisier listening situations.

Supporting Visual Access to Language

Number 1 – Encouraging Your Baby to Look to You

Getting your baby's attention *before* you communicate with them is an important way to help them access and follow what you are talking or signing about.

It can also let them know you are about to speak or sign to them. This way they won't miss the start of what you are saying.

By encouraging your baby to look to you before you communicate, in time they will soon learn to look more often by themselves.

Try calling their name – near and clear.

Depending on your baby's hearing levels and background noise, this might not always work. But you can use other ways to get your baby's attention too.

You can use touch. Tapping your child on their shoulder, arm or leg is a great way to get their attention.

Waving in your baby's line of vision can also be a really good visual cue to let your baby know you want to talk or sign to them.

You can also try moving your face into your baby's line of vision.

You can even bang the floor when you are near them.

If you are signing, you can try signing 'look at me' to encourage your baby to look to you.

You can also encourage your baby to look at you and your signs by placing them in front of your child.

If you are signing about what your child is looking at, you can place your signs next to it.

When encouraging your baby to look at you, it can be helpful to try a few times to get their attention using these methods.

But these attention getters won't always work...and that's okay too! All babies will sometimes just want to do their own thing.

And remember, although talking to your baby about what they are interested in and supporting their access to what you are saying or signing creates lots of great language learning opportunities, you don't need to do this all the time.

It can be really helpful for your baby if you sometimes just sit back and give them time to explore.

Supporting Visual Access to Language

Number 2 – Getting to Your Baby's Level

Getting to your baby's level makes it much easier for your child look at you and to see your lip-patterns, facial expressions, natural gestures, and signs if you are using them.

It also makes it easier for your baby to see visual cues that you are communicating or are about to communicate with them.

This is particularly important for babies with very limited hearing as they will rely more on seeing your face to access your communication.

When getting to your baby's level, try to get in just the right position so that your child can easily see your face and can easily switch between looking at what they're playing with and looking at you.

Your baby will also still have good auditory access if they are using hearing technologies.

As babies grow older, they become able to follow where you are looking. This can help them to understand what you are talking about. Getting to your baby's level can help them do this.

Supporting Visual Access to Language

Number 3 – Lighting

When you can, try to make sure there is enough light on your face to help your child see you clearly. Remember, a bright window behind you can make it harder for your baby to see your face.

Finding the Balance

This section looks at how to provide the best access to language in everyday situations for *your* baby.

It might be helpful to pause the video and make some notes as we go along.

Let’s start by thinking about your baby’s hearing levels.

- What is their current hearing loss level?
- And is this level changing?

Thinking about the answers to these questions can help you find the right balance for your baby. For example, if your baby has limited hearing you will need lean more heavily on supporting visual access to language.

Now let’s think about how your baby responds to you.

- Do they often respond more to your auditory cues or to your visual cues?

If they often respond to auditory cues when you are talking to them, you may not need to rely as much on visual tips.

It can also be helpful to think about the situation you are in when you are communicating with your baby.

Depending on the situation, you may need to change how much you support visual access to language. For example, when it is noisy, or during times when your baby is not wearing their hearing aids like bath-time or swimming, you will need to rely more on visual access.

Finally, let’s think about your family’s communication approach.

- What communication approach are you currently using or thinking about using?
- Are you using a mix of strategies from different approaches?

Generally, for all approaches, if your baby is using hearing aids or cochlear implants, try to support their auditory access to language as much as possible.

Depending on your communication approach or approaches, you might be using specific strategies to focus on hearing and listening, so you may not need to rely as much on visual tips, but they can still be helpful.

If you are using sign, try to support your child’s visual access to language as much as possible too.

You may find that you need to focus on hearing and listening as well as visual access to language if, for example, your baby is using a cochlear implant and your family are also using sign.

How to balance this often depends on the advice your family has received from supporting professionals, how your baby responds, and the situation you are in. Sometimes, you may need to focus on supporting one more than the other.

By thinking about these things when you are communicating with your baby, you will be able to find the right balance to support your baby’s access to language in everyday situations.

Video 5: Thinking About How We Communicate

So far, we have looked at how to create lots of great communication opportunities for your baby by:

1. tuning-in to what has caught their attention
2. talking to them about it and responding to their communication
3. helping them to *notice* you are talking or signing to them...and
4. making it easier for them to access what you are saying or signing

Now we will look at *how* we can communicate, to make learning language *easier* for your baby.

When we communicate with babies, there are changes we can make to help them learn.

Here are 5 key changes you can try when communicating with your baby:

Number 1: Infant Directed Speech
Number 2: Repetition
Number 3: Acoustic Highlighting
Number 4: Pausing and Waiting
Number 5: Using Warm, Animated Facial Expressions

These are really helpful for babies with hearing loss. So let's look at them in detail.

Number 1: Using infant directed speech

One important change is to speak in a lively, exciting and child friendly way. When talking to babies, we naturally tend to slow down, use simpler sentences, emphasise words and phrases, and use a sort of sing-song voice by changing our pitch and intonation.

This is called Infant Directed Speech.

Research shows that this helps to support language development for *all* babies including babies with hearing loss, who are wearing hearing aids or cochlear implants.

It is important because when we talk this way, babies pay more attention to what we are saying. It also helps them to pick out words from our conversations with them.

If you are signing with your baby, you can make similar changes by signing slowly, making your signs bigger and placing your signs near what you are talking about.

If you talk and sign at the same time, you *can* bring these tips together.

Number 2: Using repetition

Repetition is *incredibly* valuable when talking or signing to your baby.

Sometimes, your child may miss what you have said or signed but repetition gives them more opportunities to see and hear you.

It also gives them time to process what you have said or signed.

There are lots of natural opportunities for repetition in everyday situations like mealtimes, nappy changing, or even going up and down the stairs.

Number 3: Using acoustic highlighting

To help your baby learn a specific word, it can sometimes help if you stress the word by saying it a little slower or a little louder than the rest of your sentence, so that it stands out.

Making it easier for your baby to identify a word will make it easier to learn.

It can also help to say that specific word at the end of your sentence. It can be easier for babies to learn a word if it is the last word they heard.

Number 4: Pause and wait

When speaking or signing to your baby, pausing from time to time can help, as it gives your baby a little more time to process what you have said or signed. It also gives your baby an opportunity to respond and take turns with you.

When pausing and waiting, it can help to wait longer than you normally would with an adult. It might sometimes feel like a long time, but it won't for your baby.

Number 5: Using facial expressions

Using big, warm, positive facial expressions and body language when speaking or signing to your baby, is rewarding for them and encourages them to pay attention to what you are saying or signing.

It can also help your baby to recognise your reactions and emotions.

You can also use facial expressions and gestures to support what you are saying.

You can make these 5 adaptations in everyday situations to make the way you talk or sign engaging, and easy for your baby to understand.

It's all about increasing the number of opportunities they have to learn to communicate.

Remember! When communicating with and responding to your baby, the key is to provide the best possible access to language for them in everyday situations and to give them as many opportunities as possible to communicate.

Babies might use lots of different ways to try to communicate with you. Through sight, sound, touch and more.

Generally speaking, responding to *anything* that gets communication off the ground is really helpful. It's all about connecting with your baby.
